# The Semi-Penalized Updated Properties Model and Its Algorithm to Impose the Volume Fraction

**DOI:** 10.3390/ma18132972

**Published:** 2025-06-23

**Authors:** Amin Alibakhshi, Luis Saucedo-Mora

**Affiliations:** E.T.S. de Ingeniería Aeronáutica y del Espacio, Universidad Politécnica de Madrid, Pza. Cardenal Cisneros 3, 28040 Madrid, Spain; amin.alibakhshi@upm.es

**Keywords:** topology optimization, updated properties models, density-dependent Young modulus, volume constraint

## Abstract

Intricate structures with minimal weight and maximum stiffness are demanded in many practical engineering applications. Topology optimization is a method for designing these structures, and the rise of additive manufacturing technologies has opened the door to their production. In a recently published paper, a novel topology optimization algorithm, named the Updated Properties Model (UPM), was developed with the homogenization of strain level as an objective function and an updating Young modulus as the design variable. The UPM method optimizes mechanical structures without applying any constraints. However, including constraints such as volume, mass, and/or stress in topology optimization is prevalent. This paper uses the density-dependent Young modulus concept to incorporate the volume fraction in the UPM method. We address the critical problem of constraint-aware design without the complexity of constraint-handling formulations. We show the proposed methodology’s success and functionality by plotting the algorithm’s results in two- and three-dimensional benchmark structures. Key results present that adjusting algorithmic parameters can yield both binary (single-material) and graded-material solutions, offering flexibility for different applications. These findings suggest that the UPM can effectively replicate constraint-driven outcomes without explicitly enforcing constraints. The main novelty of this work lies in extending the constraint-free UPM framework to allow for controlled material distribution using a physically meaningful update rule. This extends the applicability of the UPM beyond previous efforts in the literature. We have also created a Julia package for our proposal.

## 1. Introduction

Topology optimization is a practical mathematical approach for optimizing structural layouts within a design domain, considering specified load conditions, boundary constraints, and performance objectives [1,2]. Since the introduction of the seminal paper by Bendsøe and Kikuchi [3], the development of topology optimization methodologies has been a popular topic of interest for researchers and designers.

Over time, numerous methods have been introduced in topology optimization to enhance its efficiency in solving complex problems and expand its applications to various domains, including mechanical components [4,5,6], electromagnetics [7,8,9], and photonics [10,11,12], among others. The homogenization design method (HDM) [13,14,15], based on the homogenization approach, the solid isotropic material with penalization (SIMP) method [16,17,18], the level set method [19,20,21], the evolutionary structural optimization (ESO) method [22,23,24], and the moving morphable components (MMC) method [25,26,27], to name a few, are several topology optimization techniques that are commonly utilized both in academic and industrial settings.

Since the publication of the 99-line MATLAB code [28] for topology optimization, recognized as an educational paper, numerous studies have been dedicated to developing topology optimization codes in various programming languages, including MATLAB and Python [29,30,31,32,33]. In addition, topology optimization methods have been implemented for both single- and multimaterial topology optimization [34]. The Alternating Active-Phase Algorithm [35], Ordered SIMP [36], and Level-Set method [37] are considered some of the most prevalent techniques for multimaterial topology optimization. Through topology optimization, various problems have been addressed, including—but not limited to—imperfect structures under load position uncertainty [38], the plastic-limit behavior of I-beams [39], and structural performance enhancement under realistic constraints [40].

In most studies on topology optimization, the objective function is typically defined as minimizing compliance or, equivalently, maximizing stiffness with volume or mass constraints. Other types of constraints, such as stress [41,42,43], displacement [44,45], manufacturing [46,47,48], and buckling constraints [49,50,51], have also been introduced, among others. In topology optimization, a binary solution is usually obtained, which is shown by the relative density, namely the design variable. Various methods have been introduced to achieve an optimum topology optimization solution, classified as gradient-based and gradient-free methods.

Gradient-free algorithms include the Genetic algorithm [52], particle swarm optimization [53], simulation annealing [54], and Nelder–Mead simplex [55], which are well suited for nondifferentiable functions, mixed design variables, discrete feasible space, and disconnected feasible space [56].

In contrast, gradient-based algorithms deal with the gradient of objective functions and constraints. For example, Sigmund introduced the optimality criteria (OC) [28] that uses the Karush–Kuhn–Tucker first-order optimality condition and Kim et al. [56] introduced its generalized form. Furthermore, Sequential Linear Programming (SLP) and Sequential Quadratic Programming (SQP) [31,57,58] are other alternative algorithms for topology optimization. The method of moving asymptotes (MMA), used for large-scale problems, is another approach developed by Svanberg [59], which has been widely used for stress-constrained topology optimization [58].

A newly introduced approach, the Updated Properties Model (UPM) [60,61], offers a novel optimization algorithm. In this approach, the objective function is to minimize the standard deviation of the differentiation of the strain energy with respect to a material parameter, such as the Poisson ratio or Young modulus, with the latter being used in the original version. The strain field is obtained by differentiating the strain energy and, through the iterative updating of Young’s modulus as a design variable, the strain at the micro-level (element strain) is homogenized, leading to stiffness maximization. In addition, this objective function has been shown to result in a reduction in compliance. In contrast to typical topology optimization algorithms, where the design variable is the density, the UPM iteratively updates the Young modulus as the design variable instead. This is favorable for multiscale topology optimization and metamaterial generation, achieved by controlling the Young modulus (stiffness) at a micro resolution.

An additional benefit of the UPM method is that it does not require information on the Hessian matrix, namely second-order differentiation, which is computationally expensive and requires significant memory allocation [56]. This benefit is also seen in the aforementioned algorithms. Moreover, in the UPM method, there are no constraints except for static equilibrium, which is satisfied automatically. Furthermore, similar to the SIMP method, the side constraints, namely the upper and lower bounds for the Young modulus, are satisfied directly when the design variables are obtained.

The UPM method does not explicitly enforce constraints such as volume or mass. However, in many practical applications, it is essential to control the amount of material used in optimized structures. This raises the following question: how can material distribution be guided in a constraint-free framework? In this work, we address this by introducing a semi-penalized formulation of the Updated Properties Model (UPM), which incorporates a density-dependent Young modulus combined with a transformation function. This approach allows the designer to control volume fraction without directly formulating or differentiating constraint terms, unlike classical methods such as SIMP.

The results show that the method can produce both binary (single-material) and functionally graded material (FGM) distributions, with comparable or improved mechanical performance relative to conventional approaches. Importantly, the FGM structures generated by our method exhibit non-predefined spatial patterns, in contrast to many traditional approaches [62], which assume specific mixing laws or directionally controlled gradients.

FGMs are highly important in applications requiring tailored stiffness, thermal, or acoustic properties. Previous non-predefined FGM approaches often rely on mixture-based modeling [63,64] and have more than one design variable to control FGM patterns. In contrast, our formulation introduces a new design variable (Young modulus) and a new update law, offering a physically grounded alternative that supports a broader and more flexible material design space.

## 2. Materials and Methods

In this section, we derive the topology optimization formulation for the UPM method with a volume constraint. We define the topology optimization problem in the UPM method as

(1) $$\left\{\begin{array}{c} \min_{H}\;s_{H}\left(H\right):=\sqrt{\frac{1}{N}\sum_{e=1}^{N}{\left(H_{e}-\bar{H}\right)}^{2}},\\ \;s.t.\;ku=f,\\ \;E_{\min}\leq E_{e}\leq E_{\max},\;e=1,\ldots ,N. \end{array}\right.$$

in which $H={\left\{H_{1},H_{2},H_{3},\ldots ,H_{N}\right\}}^{T}$ stands for the vector of the design variables that is an energetic measurement of the strain level $H_{e}=\partial \Psi_{e}/\partial E_{e}\Omega_{0},i=1,2,\ldots ,N$, $s_{H}$ shows the standard deviation of H, $\bar{H}$ represents the the mean of H, *N* refers to the number of the element, $\Omega_{0}$ is the element volume, and $\Psi_{e}$ stands for the element strain energy.

We utilize the finite element method (FEM) to discretize the design domain. In order to define the strain energy and its differentiation equation, we use the Principle of Virtual Work as

(2) $$\delta W_{int}=\delta W_{ext}$$

or equivalently

(3) $$\int_{\Omega }\sigma :\delta \varepsilon \;d\Omega =\int_{V}b\cdot \delta u\;d\Omega +\int_{S}t\cdot \delta u\;dS+\sum_{i}u_{i}^{T}R_{i}$$

in which Ω exhibits the volume of the body; *S* indicates the surface boundary of the body; the Cauchy stress tensor is shown by σ; ε stands for the strain, obtained from the displacement u; b refers to the body force per unit volume; t is the traction per unit area on the boundary; and *R* represents the concentrated force.

Solving the static equilibrium equation ku=f, we can obtain the displacement and strain that result in obtaining the strain energy as

(4) $$\Psi_{e}=\frac{1}{2}\int_{\Omega_{0}}\varepsilon^{T}D_{e}\varepsilon \;d\Omega_{0}=\frac{1}{2}u_{e}k_{e}u_{e}$$

where $D_{e}$ is the rank-4 elasticity tensor. The total strain energy is obtained by the summation of the strain energy of all elements, expressed as $W=\sum_{e=1}^{N}\Psi_{e}$.

The quantity H for each element is then derived as

(5) $$H_{e}=\frac{1}{2\Omega_{e}}\int_{\Omega_{0}}\varepsilon^{T}{\hat{D}}_{e}\varepsilon \;d\Omega_{0}$$

with ${\hat{D}}_{e}=D_{e}/E_{e}$.

To find the local minimum of the objective function in Equation (1) for the given design variables, we use Newton’s method of optimization, such that

(6) $$H^{t+1}=H^{t}-{\left[\nabla \nabla s_{H}\right]}^{-1}\left(\nabla s_{H}\right)$$

As reported in [61], the Hessian $\nabla \nabla s_{H}$ is not invertible, and we cannot use Newton’s method to find the local minimum directly. For this reason, we use gradient descent to find the updating formula as follows

(7) $$H_{e}^{t+1}=H_{e}^{t+1}-\eta_{e}^{t}\nabla s_{H}$$

with

(8) $$\nabla s_{H}=\frac{H_{e}-\bar{H}}{Ns_{H}}$$

Using the following relationship

(9) $$\eta_{e}=H_{e}N$$

and

(10) $$\Psi_{e}=E_{e}H_{e}\upsilon_{0}$$

we obtain

(11) $$\frac{\Psi_{e}^{t+1}}{E_{e}^{t+1}}=\frac{\Psi_{e}^{t}}{E_{e}^{t}}\left(1-\frac{H_{e}^{t}-\bar{H^{t}}}{s_{H}^{t}}\right)$$

Assuming sufficiently slow updating, we can write $\Psi_{e}^{t+1}≃\Psi_{e}^{t}$, resulting in

(12) $$E_{e}^{t+1}=\frac{E_{e}^{t}}{1-\alpha_{e}^{t}}$$

in which

(13) $$\alpha_{e}^{t}=\frac{H_{e}^{t}-\bar{H^{t}}}{s_{H}^{t}}$$

In Equation (12), we have a nonlinear updating formula in terms of $\alpha_{e}$, which can be more simplified using the first term of the Taylor series expansion if we assume that α is a small parameter, such that

(14) $$E_{e}^{t+1}=\frac{E_{e}^{t}}{1-\alpha_{e}^{t}}=E_{e}^{t}\left(1+\alpha_{e}^{t}\right)$$

Now, we can obtain the final updating formula as

(15) $$E_{e}^{t+1}=E_{e}^{t}\left(1+\frac{H_{e}^{t}-\bar{H^{t}}}{ks_{H}^{t}}\right)$$

where *k* is a tuning parameter to the magnitude of the step taken. As we can see, the new procedure used to obtain the minimum of the objective function does not require a Hessian Matrix. Furthermore, we use Young’s modulus as the design variable instead of H, which simplifies the topology optimization process. The formulation above and the updating formula in Equation (15) comes from the original UPM method, which does not include any constraints. In what follows, we show a strategy for applying the volume constraint.

We use the density-dependent Young modulus scheme, a transformation function, and a bisection search to apply volume fraction. After updating the Young modulus at each iteration, it is transformed to the density based on the density-dependent formulation of the Young modulus as [65]

(16) $$E\left(\rho \right)=E_{0}{\left(\frac{\rho }{\rho_{0}}\right)}^{\gamma }$$

wherein $E_{0}$ and $\rho_{0}$ represent the reference Young modulus and material density, respectively; and γ stands for a given exponent. The density vector obtained from Equation (16), $\rho ={\left\{\rho_{1},\rho_{2},\rho_{3},\ldots ,\rho_{N}\right\}}^{T}$, represents the raw density without enforcing the volume constraint. To impose this constraint, we introduce the following transformation function

(17) $$T_{v_{f}}\left(\rho_{i},\rho_{\mathrm{tr}},\eta \right)=\left\{\begin{array}{cc} \;\rho_{\min} & \mathrm{if}\;\rho_{i}<\rho_{\mathrm{tr}}\\ \tan\left(\eta \right)\cdot \left(\rho_{i}-\rho_{\mathrm{tr}}\right) & \mathrm{if}\;\rho_{\mathrm{tr}}\leq \rho_{i}\leq \rho_{\mathrm{tr}}+\frac{1}{\tan(\eta )}\\ \;\rho_{\max} & \mathrm{if}\;\rho_{i}>\rho_{\mathrm{tr}}+\frac{1}{\tan(\eta )} \end{array}\right.$$

that should satisfy

(18) $$\frac{1}{N}\sum_{i=1}^{N}T_{v_{f}}\left(\rho_{i},\rho_{tr},\eta \right)=v_{f}$$

In Equations (17) and (18), η adjusts the sharpness of the transition between $\rho_{min}=0.01$ and $\rho_{max}=1$, the limit of the real density, $\rho_{tr}$ shows the threshold density, which is obtained with the help of the bisection method, and $v_{f}$ represents the volume fraction. Since the transformation function alters the density distribution, the threshold density $\rho_{tr}$ must be iteratively adjusted to achieve the target volume fraction. Consequently, we define an initial bound for the threshold density as $\rho_{vf0,\mathrm{bound}}=-1/tan\left(\eta \right)$ and $\rho_{vf1,\mathrm{bound}}=1$, which stand for the threshold at which the volume fraction becomes 0 and 1. We consider an initial large error value to ensure that the iterative process starts; here, we assume that the error is error=10.0. The bisection search starts with midpoint search as $\rho_{tr}=\left(\rho_{vf0,\mathrm{bound}}+\rho_{vf1,\mathrm{bound}}\right)/2$, and then we derive the transformed sum at the upper, lower, and midpoint bounds as

(19) $$\begin{array}{cc} \mathrm{Upper}\;\mathrm{bound}:\;S_{\mathrm{vf}1} & =\frac{1}{N}\sum_{i=1}^{N}T\left(\rho_{i},\rho_{\mathrm{vf}1,\;\mathrm{bound}},\eta \right)\\ \mathrm{Lower}\;\mathrm{bound}:\;S_{\mathrm{vf}0} & =\frac{1}{N}\sum_{i=1}^{N}T\left(\rho_{i},\rho_{\mathrm{vf}0,\;\mathrm{bound}},\eta \right)\\ \mathrm{Midpoint}:\;S_{\mathrm{mid}} & =\frac{1}{N}\sum_{i=1}^{N}T\left(\rho_{i},\rho_{\mathrm{tr}},\eta \right) \end{array}$$

At this stage, the bounds can be updated as

(20) $$\rho_{\mathrm{vf},\;\mathrm{bound}}=\left\{\begin{array}{cc} \rho_{\mathrm{tr}}, & \mathrm{if}\;\frac{\left(S_{\mathrm{vf}1}-vf\right)}{\left(S_{\mathrm{mid}}-vf\right)}>0,\;\mathrm{update}\;\rho_{\mathrm{vf}1,\;\mathrm{bound}}\\ \rho_{\mathrm{tr}}, & \mathrm{if}\;\frac{\left(S_{\mathrm{vf}0}-vf\right)}{\left(S_{\mathrm{mid}}-vf\right)}>0,\;\mathrm{update}\;\rho_{\mathrm{vf}0,\;\mathrm{bound}}\\ \mathrm{Error}:\;\mathrm{Out}-\mathrm{of}-\mathrm{bounds}\;\mathrm{condition}, & \mathrm{otherwise} \end{array}\right.$$

An convergence criterion for the bisection search is also defined as $|vf-S_{\mathrm{mid}}|<0.001$. Following the steps above, the raw density is updated by enforcing the volume constraint. The convergence behavior of our method is indeed influenced by the choice of parameter η, which plays a remarkable role in the transformation function that links the density and the updated Young modulus. In our testing, we observed that if η is not selected appropriately, the optimization process may fail to converge as early as the first iteration. A numerical error typically accompanies this behavior. Specifically, we found that convergence is robust and reliable when η is chosen within the range (π/2,π/4]. Within this interval, the method consistently converges within a reasonable number of iterations for both the bisection search and the main optimization loop. That said, it is possible that in some specific cases, especially for certain boundary conditions or loading configurations, convergence may still occur outside this recommended range. However, the range we report serves as a safe guideline based on our current testing across standard benchmarks.

In the proposed method used to enforce the volume fraction, parameter η plays a crucial role. As stated above, this parameter controls the smoothness and aggressiveness of the transformation function. When η is small, the transition region is wide, resulting in density values within the intermediate transformed range, similar to non-penalized topology optimization. As η increases, the optimized structure tends toward a binary solution, eliminating intermediate values, with densities pushed toward $\rho_{\min}$ or $\rho_{\max}$.

The general iterative process in topology optimization must converge. The convergence criterion is established based on the strain energy at each iteration, as follows:

(21) $$A=\frac{W^{t+1}-W^{t}}{W^{t}}\leq \epsilon$$

in which ϵ is a tolerance.

The following, Algorithm 1, presents a pseudocode for implementing the proposed algorithm in a programming language.
**Algorithm** **1:** Semi-penalized UPM with volume fraction**Require:** Model setup: boundary conditions (Dirichlet and Neumann) and bounding volume.

**Ensure:** *FEA isotropic linear elastic calculation at *
$\;t=0\to k{(}^{0}E)u=f$
1:Initialize parameters: $E,\;k,\;\gamma ,\;\mathrm{volfrac},\;\eta ,\;\rho_{0},\;\mathrm{maxi}\_\mathrm{tr},\;\mathrm{tol},\;E_{0}$2:Set loop counter loop=13:Compute initial FEA solution: Solve k(E)u=f, obtain compliance *C* and strain energy $W_{\mathrm{tot}}$4:Store strain energy history: $W_{\mathrm{tot}}^{\left(1\right)},W_{\mathrm{tot}}^{\left(2\right)}=10W_{\mathrm{tot}}^{\left(1\right)}$5:Compute initial convergence measure $A=\left(W_{\mathrm{tot}}^{\left(2\right)}-W_{\mathrm{tot}}^{\left(1\right)}\right)/W_{\mathrm{tot}}^{\left(1\right)}$6:Print iteration status7:**while** |A| > tol **and** loop ≤ max_itr **do**8:      Solve FEA: k(E)u=f, compute compliance *C* and total strain energy $W_{\mathrm{tot}}$9:      Update material properties: $E_{\mathrm{new}}\;=\;\mathrm{update}\_\mathrm{upm}\left(k,E,H,E_{\max},E_{\min}\right)$10:    Compute density $\rho \;=\;\mathrm{transfer}\_\mathrm{to}\_\mathrm{density}(E_{\mathrm{new}},E_{0},\rho_{0},\gamma )$11:    Filter density: $\rho_{\mathrm{new}}\;=\;\mathrm{filter}\_\mathrm{density}\_\mathrm{to}\_\mathrm{vf}\left(\rho ,\mathrm{volfrac},t_{\mathrm{nele}},\eta \right)$12:    Convert density to Young’s modulus: $E_{\mathrm{new}\_\mathrm{frac}}=\mathrm{transfer}\_\mathrm{to}\_\mathrm{young}\left(\rho_{\mathrm{new}},E_{0},\rho_{0},\gamma ,E_{\min},E_{\max}\right)$13:    Update material properties in the model: $E=E_{\mathrm{new}\_\mathrm{frac}}$14:    Solve FEA with updated material: Obtain compliance *C*, displacement *U*, stress σ, and strain ε15:    Store and export results to VTK file16:    Update strain energy history and recompute convergence measure *A*17:    Increment loop counter18:    Print iteration status19:**end while**20:**if** loop > max_itr **then**21:    **Terminate:** Maximum iterations reached, compliance is set to −122:**end if**23:**Final Computation:** Solve FEA with final material distribution and export results


## 3. Results and Discussion

In this section, we examine the efficiency of our proposal. We investigate the influence of different parameters, such as the volume fraction $v_{f}$, γ, and η. For all results, we use $\rho_{0}=1$, $E_{0}=1$, Poisson’s ratio ν=0.3, $E_{\min}=10^{-4}$, and $E_{\max}=1$.

**Figure 1:**
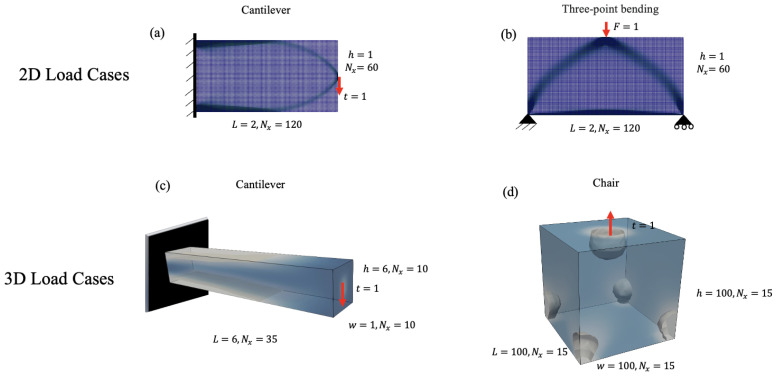
Schematic representation of the load and boundary conditions for two- and three- dimensional geometries. (**a**) 2D cantilever, (**b**) three-point bending (3pb), (**c**) 3D cantilever, and (**d**) 3D chair.

The load cases for two (2D) and three-dimensional (3D) structures are shown in Figure 1. As shown in Figure 1a, the first 2D case is a cantilever subjected to traction load at the middle of the free end of t=1 [MPa], with the length L=2[mm], the height h=1[mm] and the number of elements in the x-direction as $N_{x}=120$ and y-direction $N_{y}=60$.

The second 2D case involves a three-point bending beam (3pb) with a concentrated load of F=1 [N] applied in the upper center (see Figure 1b). The general results for the cantilever and three-point bending (3pb) cases are similar; therefore, for brevity, we present the 3pb results in Appendix A.

For the 3D case, we consider a cantilever and a chair with the same traction load. The length *L* [mm], width *w* [mm] and height *h* [mm] of these geometries are shown in Figure 1c,d. We impose a Dirichlet boundary condition for the chair on the bottom surfaces in four corner circles, each with a radius of 10 [mm]. The adopted Neuman boundary condition is a circle at the top surface with a radius of 20 [mm]. For 2D and 3D cases, we use quadrilateral and hexahedral elements, respectively.

**Figure 2:**
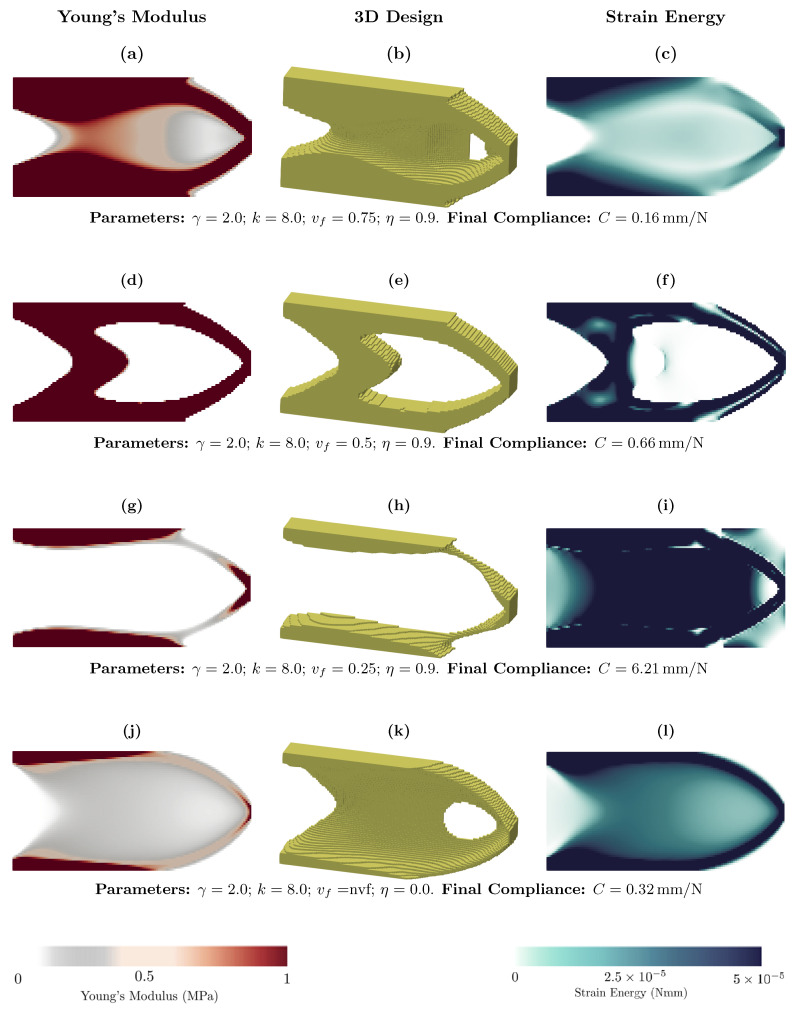
Effect of the volume fraction on topology optimization of the two-dimensional cantilever.

We show the effect of the volume fraction on topology optimization in Figure 2. We chose three values for the volume fraction $v_{f}=0.25,\;0.5,\;0.75$ and a case without volume fraction $v_{f}=nvf$, namely the original UPM method. We depict the Young modulus map (design variables), the 3D view of the optimized structure, and the strain energy. The figures show the success of the proposed method in imposing the volume fraction. From the figures, we can see that as the volume fraction decreases, less material is in the optimized structure. This means that the distribution of material within the design domain is effected by varying the volume fraction. Furthermore, we observe that a larger volume fraction in the optimized structure results in more material and reduced compliance, indicating greater stiffness compared to structures with a smaller volume fraction, which is expected.

Figure 2j–l shows the result of the cantilever without a volume fraction, which is related to the original UPM method. For this case, the optimized structure has more intermediate Young modulus values, similar to the SIMP method, with a penalty equal to the unity. Moreover, it is clear that in the UPM, we do not have any volume fraction parameter, and only one solution is obtained.

The optimized structure can be the binary solution, as can be seen in Figure 2d–f and the the other functionally graded sub-figures in Figure 2. In the graded optimized structure, soft materials with lower Young’s modulus and stiffer materials with higher Young’s modulus coexist. This combination may result in better performance compared to single-material structures by balancing softness and stiffness, ultimately reducing overall compliance.

In the proposed algorithm, the relationship between Young’s modulus and density is given by Equation (16). This equation resembles the interpolation used in the SIMP method, which incorporates a penalty parameter, typically denoted as γ. However, as shown in Figure 2, even when γ=2, intermediate density values still appear in some cases. This behavior differs from the standard SIMP method, where a penalty greater than one typically suppresses intermediate values. Therefore, γ in the proposed algorithm does not act in the same way as the penalty parameter in SIMP. As we can see in the following, when the effect of γ is analyzed, it changes the layout of the material within the design domain. Further details on the effect of γ will be presented in Figure 4.

**Figure 3:**
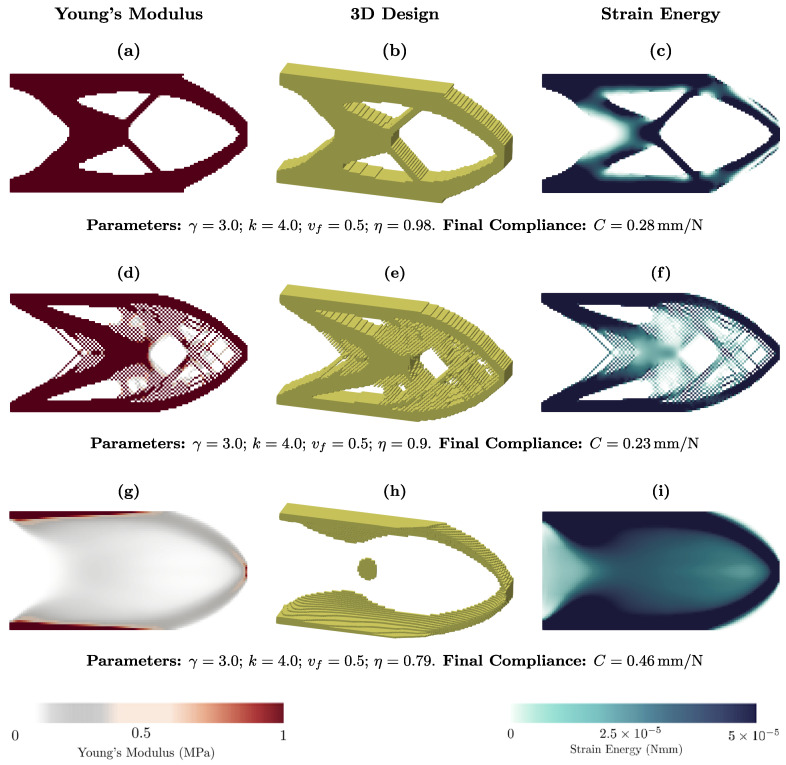
Effect of parameter η on the topology optimization of the two-dimensional cantilever.

We illustrate the influence of η on topology optimization in Figure 3 for the 2D cantilever load case. The lowest compliance is obtained for η = 0.9; however, the optimized structure exhibits checkerboard patterns. Comparing Figure 3a–c with Figure 3g–i, which show solutions without checkerboard patterns, we observe that for the larger η=0.98, the stiffness is greater than that of the other case. Figure 3a–c represents a result similar to classical topology optimization algorithms with a binary solution for the Young modulus. As this parameter decreases, the intermediate values of the design variables become predominant. Taken together, η plays a crucial role in the proposed model, as it can lead to checkerboard patterns, a binary solution, or a solution with intermediate values of Young’s modulus.

**Figure 4:**
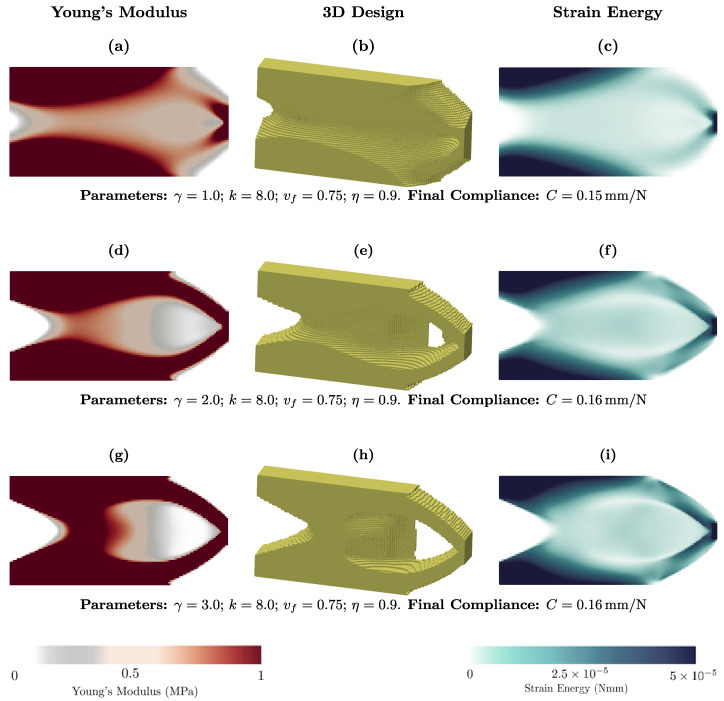
Effect of parameter γ on the topology optimization of the two-dimensional cantilever.

To explore the role of parameter γ in the optimized structure, we plot Figure 4. We observe that this parameter does not significantly affect the compliance (stiffness) for the given conditions. For γ=1.0, the optimized structure is the stiffest and, as this parameter increases, the stiffness decreases. This result is obtained because increasing γ reduces the amount of material within the design domain. It is clear that for different values of γ, the optimized structure has a graded material, with intermediate values. This exponent parameter is analogous to the penalty term in the SIMP model in terms of formulation, but it carries a different interpretation. Figure 4c,f,i shows the strain energy map in the optimized structure for different γ. In Figure 4c, the strain energy is spread more broadly within the design domain, indicating that the deformation is distributed more evenly. By increasing γ, the strain energy becomes more localized, and eventually the design becomes more refined, with a more precise separation.

**Figure 5:**
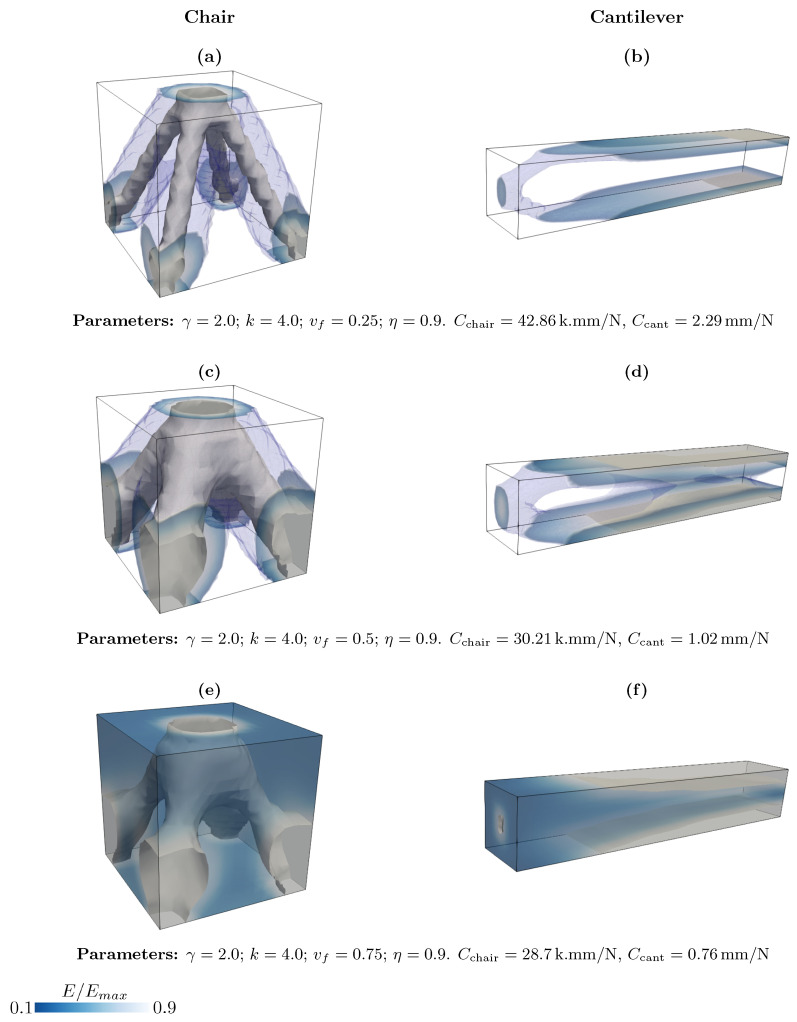
Effect of the volume fraction on the topology optimization of the three-dimensional chair and cantilever.

Here, we show the result of the topology optimization algorithm in three-dimensional structures, where there are a cantilever and a cubic that we call a chair because its optimized structure resembles a chair. Figure 5 shows the influence of the volume fraction on two 3D structures, the cantilever and chair-type loading structure. Changing the volume fraction $v_{f}$ alters the distribution of the material within the design domain, as observed. It is also clear that increasing the volume fraction leads to a denser, stronger structure, but reduces the material efficiency. We observe that the compliance of the cantilever is lower than that of the chair, indicating that it is stiffer. For the chair structure, the initial cube with the same Young modulus for each element at the first loop is updated iteratively and minimizes the energy, leading to an optimized structure with four legs, starting from the Dirichlet boundary condition to the Neumann boundary condition where the load is applied. The same scenario is also seen for the cantilever, where the material is distributed in the loading path to the fixed end of the structure, namely the Dirichlet boundary condition.

**Figure 6:**
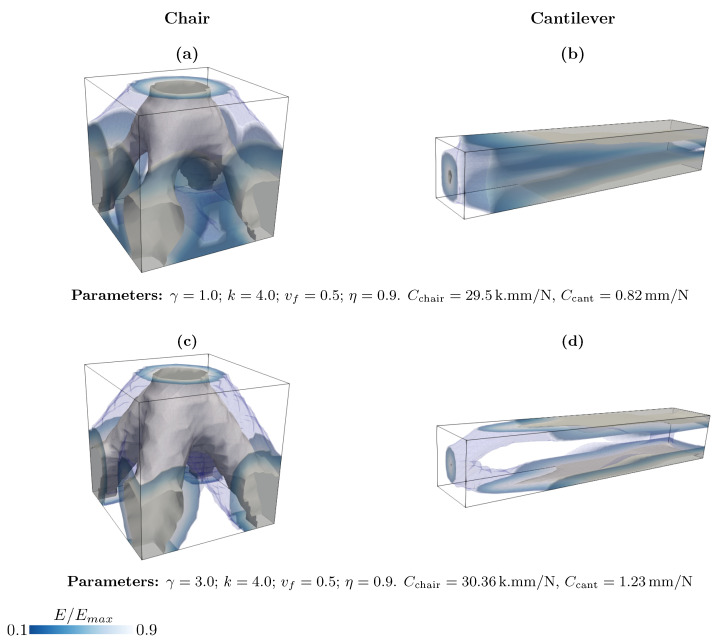
Effect of γ on topology optimization of the three-dimensional chair and cantilever.

**Figure A3:**
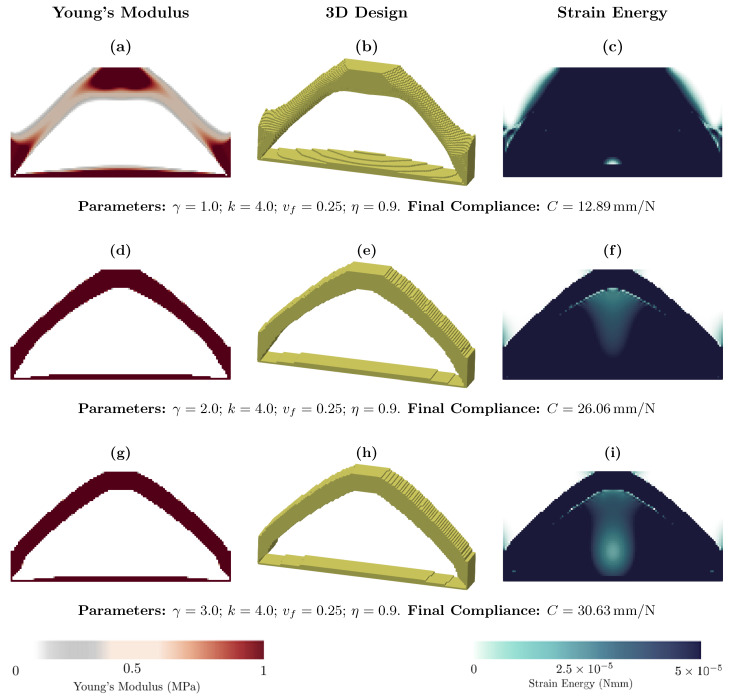
Influence of parameter γ on the topology optimization of the three-point bending (3pb).

We explore the effect of parameter γ on the topology optimization with our proposal in Figure 6. It is shown that for γ=1, the optimized structure has a more diffuse material distribution and intermediate densities. Furthermore, the compliance in this case is less than that of γ=3, which means a stiffer structure. In summary, and also based on the results in Appendix A, for the case of the 3pb load, we see that the main impact of γ is the reduction of material in the optimized structure. Furthermore, although increasing its value can help avoid intermediate densities, for example, in the 3pb case (Figure A3)—it may still preserve some intermediate values. In general, the effect of this parameter depends on the values of other parameters.

We compare the semi-penalized UPM with the SIMP method due to their similar topology optimization strategies, aiming to identify the optimal structure based on density in the case of SIMP and Young modulus for the semi-penalized UPM. We consider an MMB beam as the structure, with $N_{x}=120$, $N_{y}=60$, a penalty factor of 1, and r=1.5 for the SIMP method. The semi-penalized UPM uses the same number of elements, with dimensions L=120 and h=60 and algorithm parameters k=4,η=π/4, and γ=1.

**Figure 7:**
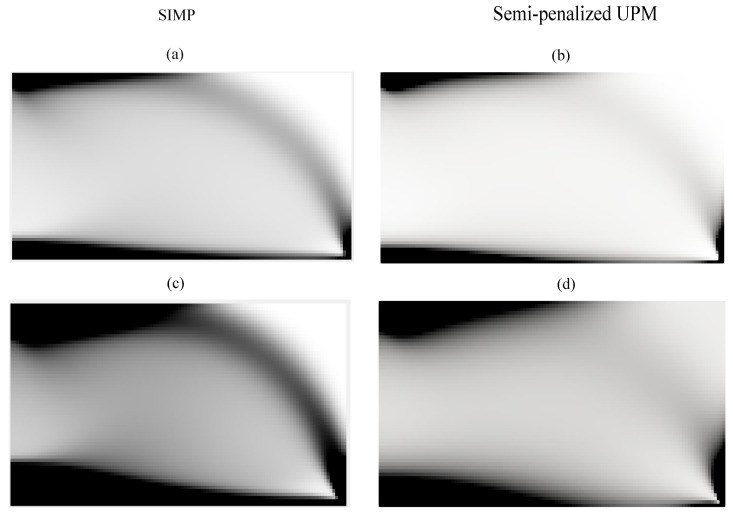
Comparison between the SIMP method and the semi-penalized UPM. (**a**) SIMP with $v_{f}=0.25$, (**b**) semi-penalized UPM with $v_{f}=0.25$, (**c**) SIMP with $v_{f}=0.5$, and (**d**) semi-penalized UPM with $v_{f}=0.5$.

Figure 7 illustrates a comparison between the SIMP method and the semi-penalized UPM in terms of their topology optimization results under different volume fractions $v_{f}$. Figure 7a depicts the result for the SIMP method with $v_{f}=0.25$, showing relatively lower material usage. In Figure 7b, the semi-penalized UPM with the same volume fraction exhibits a smoother transition between solid and void regions, possibly indicating a more graded or optimized distribution. For the cases with $v_{f}=0.5$, we also observe that the semi-penalized UPM yields a smoother and slightly more continuous material transition compared to the SIMP method

Overall, the general layout for both approaches is similar; however, the SIMP method tends to produce more binary (black-and-white) results, while the semi-penalized UPM generates smoother gradients, potentially due to its use of Young modulus as the optimization variable rather than density.

One of the key considerations in topology optimization is computational efficiency. The developed algorithm has been implemented in the Julia programming language, which is known for its speed and efficiency. To evaluate the computational performance of the semi-penalized UPM method, we compare it with the SIMP method for the cases shown in Figure 7c,d. For a fair comparison, the SIMP method was also implemented in Julia.

The computational time for the SIMP method is approximately 2.305 s, while the semi-penalized UPM completes in 3.579 s. Although this comparison is based on our specific implementation and may vary with different setups, the results demonstrate that the semi-penalized UPM remains computationally efficient. Its reasonable runtime, combined with its flexibility and constraint-free formulation, makes it a promising alternative for solving complex topology optimization problems, particularly in real-world applications with a large number of degrees of freedom.

The semi-penalized UPM offers a simpler and more intuitive approach to topology optimization compared to conventional methods. Instead of relying on artificial penalization and explicitly defined constraints, it uses Young’s modulus as the design variable, which is updated through physical relationships. This makes the method easier to implement and more grounded in real material behavior. By adjusting parameters like η and γ, it allows for smooth transitions between binary and graded materials, giving more flexibility in design. Traditional methods often struggle with issues like checkerboarding, mesh sensitivity, and gray regions, which require additional filters or regularization to fix. In contrast, the semi-penalized UPM naturally avoids or reduces these problems, leading to more stable and mesh-independent results without extra tuning.

**Figure 8:**
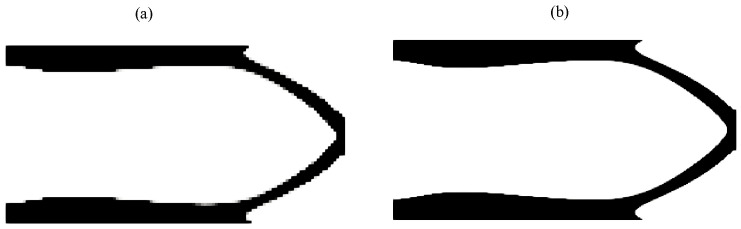
Mesh sensitivity analysis for the cantilever using the semi-penalized UPM. The algorithm parameters are γ=3, η=0.9, k=8, and $v_{f}=0.25$. (**a**) The mesh is $N_{x}=120,N_{y}=60$, and (**b**) $N_{x}=300,N_{y}=150$.

In Figure 8, we perform a mesh sensitivity analysis to demonstrate that the results of the proposed method are independent of the mesh size. Notably, this mesh independence is achieved without the use of filtering, projection methods, or regularization techniques, which are commonly required in classical approaches. This represents a key advantage of the semi-penalized UPM method. We observe that the results remain consistent across different mesh sizes; for finer meshes, the solution appears smoother, but the overall structural layout remains unchanged.

**Figure 9:**
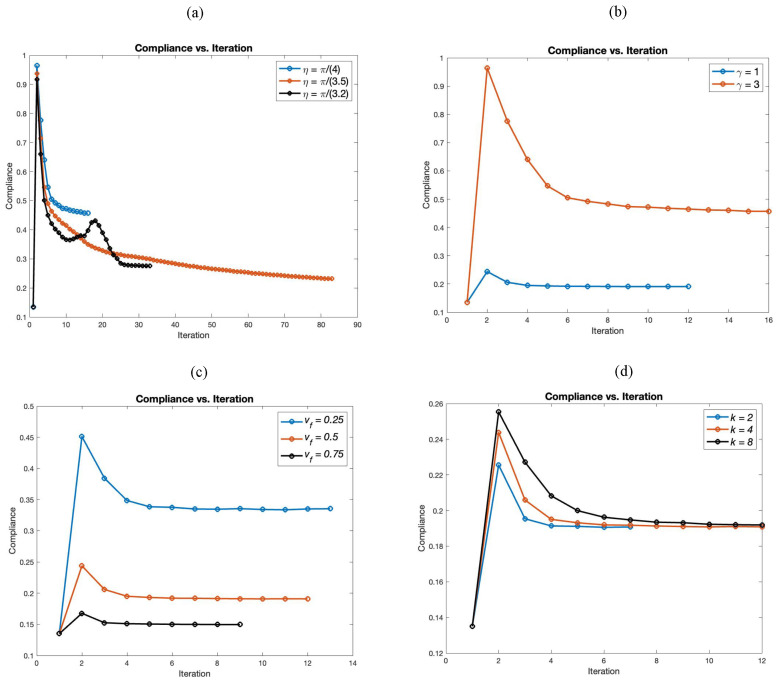
Convergence history plots: (**a**) γ=3, $v_{f}=0.5$, k=4; (**b**) η=π/4, $v_{f}=0.5$, k=4; (**c**) γ=1, η=π/4, k=4; and (**d**) γ=1, $v_{f}=0.5$, η=π/4.

Figure 9 presents the convergence history plot for the cantilever using the semi-penalized UPM. In Figure 9a, we analyze the effect of η on convergence behavior. The results show that smaller values of η lead to faster convergence but result in higher compliance (i.e., less stiffness), compared to larger values of η. As we see, η=π/(3.2) has some oscillation initially but reaches the lowest compliance eventually.

In Figure 9b, we illustrate the effect of parameter γ on the convergence history. It is observed that as γ increases, more iterations are required for convergence, and the resulting structures exhibit higher compliance, i.e., they are less stiff.

In Figure 9c, we examine the influence of the volume fraction on the convergence of the optimization. It is evident that increasing the volume fraction results in a stiffer structure and requires fewer iterations to converge compared to lower volume fractions.

Figure 9d shows the effect of parameter *k* on the compliance versus iteration curve. While the final compliance is nearly the same for all cases, the value of *k* influences the number of iterations required to reach the optimized results.

The results presented in this paper are obtained for simple and structured two- and three-dimensional geometries. Furthermore, the results are provided in image formats such as JPEG, PNG, etc. We are currently developing a Julia package to implement the proposed algorithm for more complex geometries and boundary conditions. In this package, the geometry is provided in STL or OBJ format for greater flexibility. A mesh is then generated from the geometry file, followed by the application of loads and boundary conditions. Afterward, the proposed algorithm described in this paper is applied.

One of the main challenges with complex geometries is mesh generation. To address this, we utilize the https://github.com/COMODO-research/Geogram.jl (accessed on 17 June 2025) package. The final optimized structure is exported in STL or OBJ format, making it suitable for CAD modeling and 3D printing. The package will be made publicly available in our upcoming work in the near future.

For all finite element analyses in this work, we use the open-source https://ferrite-fem.github.io/Ferrite.jl/stable/ (accessed on 17 June 2025) package, which is a flexible and efficient FEM library written in Julia. This tool provides full control over mesh generation, element formulation, and boundary condition application, and it is well-suited for research and development purposes.

The proposed topology optimization algorithm has also been implemented in our own open source Julia package in the following link: https://github.com/Aminofa70/PUPM.jl (accessed on 17 June 2025). This package directly integrates with Ferrite.jl to perform topology optimization using the semi-penalized UPM approach described in the paper. All components of our implementation are open access and reproducible, and the use of Julia offers both high computational performance and clear mathematical syntax, making it ideal for fast prototyping and scientific computing.

## 4. Conclusions

In this paper, we developed a topology optimization algorithm based on the UPM method and the density-dependent Young modulus concept. The presented algorithm develops the original UPM method by incorporating the volume constraint. The proposed method has three parameters that affect the results of topology optimization; they are the volume fraction $v_{f}$; η, the controlling parameter of sharpness of the transition between the minimum and maximum density; and γ, an exponent. We have not included any filters in the topology optimization algorithm. The semi-penalized UPM in this work can result in a binary pattern (single material) or solutions with intermediate Young modulus values. The results validate the success and operational efficiency of the developed strategy for the volume constraint. Increasing γ in the model reduces the materials and leads to greater flexibility and less stiffness. Parameter η controls the trade-off between binary and intermediate solutions as follows: increasing η promotes binary values, while decreasing it results in more intermediate values. The algorithm developed can provide a reference for the optimization of the topology of mechanical structures and be advantageous in the generation of single, FGM materials and metamaterials.

While the mathematical form of the Young modulus–density relationship in our method is similar to SIMP, the physical interpretation and design flexibility are notably different. In SIMP, the penalization forces binary solutions and suppresses intermediate densities. In contrast, our method uses a parameter γ, which plays a role similar to the SIMP penalty but enables both binary and graded designs—even when γ > 1. This behavior is not observed in penalized SIMP, where intermediate densities are typically eliminated. Additionally, our semi-penalized UPM is computationally efficient due to its constraint-free formulation and direct update rule, making it attractive for practical and large-scale applications. We do not claim that our method outperforms all others in every aspect, but we believe it offers a promising and lightweight alternative to traditional topology optimization approaches, especially in contexts where simplicity, flexibility, and computational cost are important considerations.

The semi-penalized UPM has demonstrated its effectiveness in generating optimized structures using both single materials and functionally graded materials (FGMs). However, there are still some challenges the method faces. The current formulation for the relationship between Young modulus and density is valid for linear elastic materials. Similarly, the finite element analysis employed is also based on linear elasticity. This raises important questions about extending the proposed method to nonlinear elastic materials and how to appropriately model the relationship between the Young modulus and density in such cases. Moreover, the inclusion of additional constraints, such as stress or displacement constraints, within the proposed algorithm presents another challenge that will be addressed in our future work. A critical unresolved aspect is the manufacturing of FGM structures as the output of the algorithm. Since the pattern for FGM is not predefined and shows a mixture, arranging novel configurations in manufacturing machines for such cases requires deep investigations. These cutting-edge manufacturing techniques would significantly develop structures for various applications, from aerospace to medical, where having FGMs improves the performance of structures.

## Figures and Tables

**Figure A1:**
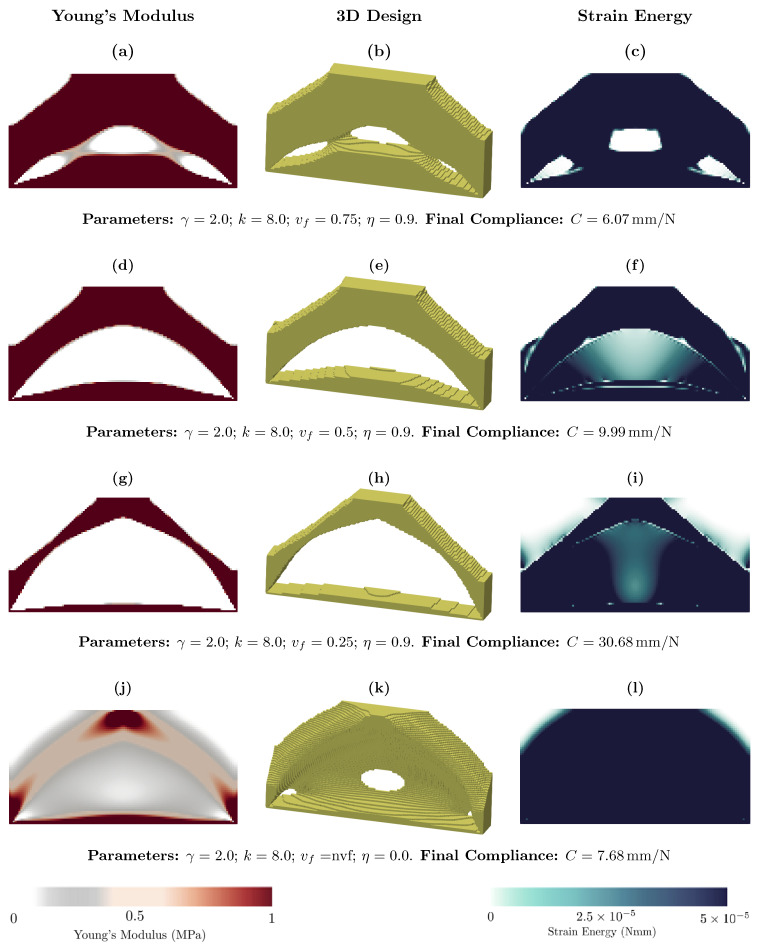
Influence of volume fraction $v_{f}$ on the topology optimization of the three-point bending (3pb).

**Figure A2:**
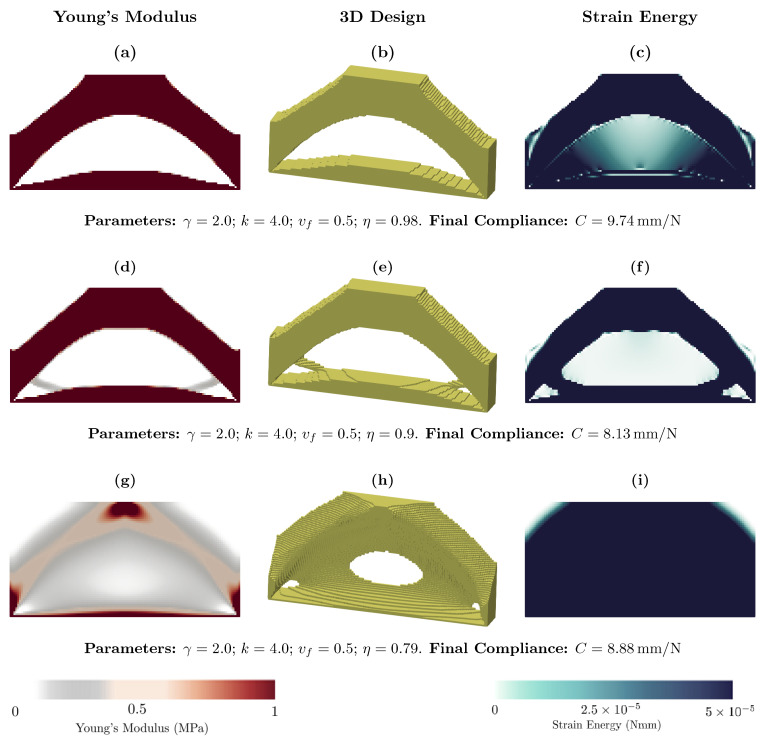
Influence of parameter η on the topology optimization of the three-point bending (3pb).

## Data Availability

The original contributions presented in this study are included in the article. Further inquiries can be directed to the corresponding author.

## Appendix A. Topology Optimization Results for 3pb

The second 2D structure in this paper is a three-point bending, (3pb), and its boundary conditions and load are shown in Figure 1b. Like the cantilever structure, we investigate the influence of $v_{f}$, η, and γ on the optimization of the topology of the 3pb topology. In Figure A1, we show how the volume fraction changes the distribution of the material within the design domain of a 3pb. As observed, similar to the cantilever beam, the volume fraction parameter effectively distributes the material. We obtain the intermediate values of Young’s modulus for the given parameters, for the original UPM method, namely, without the volume fraction, as shown in Figure A1j. For all cases, the distribution of the material is the path of the concentrated force, the top center, and the supports in the bottom corners, following the trend in the classical methods. Here we can see that we can arrive at the binary solution with γ=2, showing its difference from the penalty in the SIMP method.

The influence of η on the optimum design of the 3pb is illustrated in Figure A2. We observe that by increasing η, a denser structure with fewer voids is obtained, while decreasing this parameter results in a more complex design. For η=0.79, we obtain significant porosity and material redistribution within the design domain. We show that the greater the compliance/flexibility (that is, the lower the stiffness) for the case of η=0.98, the easier the structure deforms when subjected to applied loads. The optimized structures change with η, moving from a denser material distribution (higher η) to a perforated structure (lower η).

To demonstrate the impact of parameter γ, we plot Figure A3. For γ=1, Figure A3a, the 3pb structure has the lowest compliance *C* = 12.89 mm/N, resulting in the highest stiffness among the others. Increasing this parameter makes the structure lighter and less stiff. When increasing γ, fewer materials are seen, and there is a decrease in stiffness. We see that in the optimized structure, the material distribution changes in a graded arrangement, that is, an FGM material is obtained. As can be appreciated from Figure A3, with changing γ, we can control the pattern in the optimization, and graded materials or single materials (the binary solution) are obtained.
